# Microstructural brain tissue changes contribute to cognitive and mood deficits in adults with type 2 diabetes mellitus

**DOI:** 10.1038/s41598-023-35522-9

**Published:** 2023-06-14

**Authors:** Bhaswati Roy, Sarah E Choi, Matthew J. Freeby, Rajesh Kumar

**Affiliations:** 1 Department of Anesthesiology, David Geffen School of Medicine at UCLA, University of California Los Angeles, 56-141 CHS, 10833 Le Conte Ave, Los Angeles, CA 90095-1763 USA; 2 UCLA School of Nursing, University of California Los Angeles, Los Angeles, CA USA; 3 Department of Medicine, David Geffen School of Medicine at UCLA, University of California Los Angeles, Los Angeles, CA USA; 4 Department of Radiological Sciences, David Geffen School of Medicine at UCLA, University of California Los Angeles, Los Angeles, CA USA; 5 Department of Bioengineering, David Geffen School of Medicine at UCLA, University of California Los Angeles, Los Angeles, CA USA; 6 Brain Research Institute, University of California Los Angeles, Los Angeles, CA USA

**Keywords:** Diabetes, Translational research

## Abstract

Type 2 diabetes mellitus (T2DM) patients show brain tissue changes in mood and cognitive regulatory sites, but the nature and extent of tissue injury and their associations with symptoms are unclear. Our aim was to examine brain tissue damage in T2DM over controls using mean diffusivity (MD) computed from diffusion tensor imaging (DTI), and assess correlations with mood and cognitive symptoms in T2DM. We collected DTI series (MRI), mood, and cognitive data, from 169 subjects (68 T2DM and 101 controls). Whole-brain MD-maps were calculated, normalized, smoothed, and compared between groups, as well as correlated with mood and cognition scores in T2DM subjects. Type 2 diabetes patients showed altered cognitive and mood functions over control subjects. Multiple brain sites in T2DM patients showed elevated MD values, indicating chronic tissue changes, including the cerebellum, insula, and frontal and prefrontal cortices, cingulate, and lingual gyrus. Associations between MD values and mood and cognition scores appeared in brain sites mediating these functions. Type 2 diabetes patients show predominantly chronic brain tissue changes in areas mediating mood and cognition functions, and tissue changes from those regions correlate with mood and cognitive symptoms suggesting that the microstructural brain changes may account for the observed functional deficits.

## Introduction

Type 2 diabetes mellitus is a chronic metabolic disorder, leading to multi-systemic impairments, including the brain with well-known vascular complications, cognitive, and mood dysfunction. Epidemiologic studies have shown that individuals with type 2 diabetes have an almost 1.5-fold higher incidence of cognitive deficits^1^ and a two-fold higher risk of mood disorders^2,3^ Type 2 diabetes mellitus has been recognized as an essential risk factor for early vascular dementia and Alzheimer’s disease^4^ Earlier studies suggest that the underlying mechanisms and risk factors for early dementia and Alzheimer’s disease are overlapping in type 2 diabetes patients^5^ and such risk factors are 2-fold higher in type 2 diabetes over healthy individuals^6,7^

The relationships between type 2 diabetes mellitus and mood, including depression and anxiety have been observed in several studies^2,8^ and these patients have an increased risk of mood dysfunction compared to people without diabetes. Depression in type 2 diabetes mellitus presents a major clinical challenge with worsening diabetes self-management skills, quality of life, incidence of complications, and life span^9^ In turn, these issues may increase type 2 diabetes treatment cost for patients with symptomatic or asymptomatic depression symptoms^10^ In addition, type 2 diabetes patient exhibit clinical and subclinical anxiety more frequently than the general population. Previous studies show link between anxiety and type 2 diabetes, with prevalence rates of clinically significant anxiety up to 55% in type 2 diabetes^11,12^ Also, anxiety symptoms are associated with poor metabolic outcomes and increased medical complications in this condition.

Cognitive and mood deficits in type 2 diabetes patients can be mediated through tissue changes in regulatory sites, and brain imaging studies may help identify markers of risk for psychological comorbidities including cognitive dysfunction, depression, and anxiety and clarify cerebral pathologies in type 2 diabetes condition. Subtle brain tissue abnormalities cannot be detected with regular (commonly used) structural magnetic resonance imaging (MRI) due to insufficient sensitivity to tissue changes, but can be studied using advanced techniques such as diffusion tensor imaging (DTI), a non-invasive technique that shows changes in the microstructural organization of the brain, including gray and white matter. Mean diffusivity (MD), which measures the average movement of water molecules and represents microstructural integrity, can be calculated from DTI. MD procedures distinguish acute from chronic stages of tissue change after hypoxia/ischemia, with decreased values in acute stages, and increased values in chronic stages, and can reveal the extent and nature of the tissue injury in type 2 diabetes condition.

Most previous studies of type 2 diabetes using DTI have used fractional anisotropy measurements, which are nonspecific for acute and chronic tissue changes, to assess microstructural tissue integrity of the whole brain or specific regions of white matter tracts^13–15^ In addition, previous MD studies have shown mixed brain abnormalities in patients with type 2 diabetes^14,16^ which may be due to limited sample sizes, differences in data acquisition parameters and analysis methods. Also, direct associations between brain tissue changes in sites that mediate cognition and mood based on MD and mood and cognitive symptom scores have not been documented in type 2 diabetes patients.

In this study, we sought to examine microstructural tissue changes in patients with type 2 diabetes using whole-brain DTI based MD procedures compared to healthy controls, and assess associations between tissue integrity in mood and cognitive regulatory areas and depression, cognition, and anxiety scores in type 2 diabetes patients. We hypothesized that type 2 diabetes patients show altered MD values in mood and cognition regulatory areas compared to healthy control subjects, and tissue integrity of these areas will be associated with cognition and mood symptom scores in type 2 diabetes patients.

## Results

### Demographics, clinical, mood, and cognitive variables

Demographic, physical, and clinical data are outlined in Table 1. No significant differences in age (*p*=0.24), sex (*p*=0.62), and handedness (*p*=0.64) appeared between type 2 diabetes patients and healthy control subjects (Table 1). Body-mass-index (*p*<0.001) and systolic blood pressure (*p*=0.003) values were significantly higher in type 2 diabetes patients over control subjects. All type 2 diabetes patients were on oral diabetes medications with 17% also on insulin. Of 68 type 2 diabetes patients, 6 patients had retinopathy, 13 had neuropathy, and 7 had diabetes kidney disease. In addition, one patient had cerebrovascular disease, 3 patients had cardiovascular disease, and 2 had peripheral vascular disease. Type 2 diabetes patients had significantly higher depression [increased BDI-II] (*p*<0.001) and greater anxiety [increased BAI] (*p*<0.001) scores over healthy controls. In addition, global MoCA scores were significantly reduced in type 2 diabetes patients compared to controls (*p*=0.002), with significant differences observed in visuospatial (*p*=0.01), attention (*p*=0.005), and language cognitive subdomains (*p*=0.002).

**Table 1:** Demographics and other variables of type 2 diabetes patients and control subjects.

Variables	T2DM n = 68 (Mean ± SD)	Controls n = 101 (Mean ± SD)	*P* values
Age (years)	56.3 ± 7.7	55.0 ± 6.4	0.24
Sex [male] (%)	37 (54%)	51 (50%)	0.62
BMI	29.8 ± 5.0	26.0 ± 4.1	<0.001
Handedness [L/R/ambidex]	(n=59) [7/49/3]	(n=93) [7/82/4]	0.64
Education level (years)	15.8 ± 2.1	16.4 ± 2.7	0.14
Ethnicity/ Race	White, 21 (31%); Hispanic, 23 (34%); African American 5 (7%); Asian, 11 (16%); American Indian 3 (4%); Pacific Islander 1 (1%) and Others, 4 (6%)	White, 38 (38%); Hispanic, 21 (21%); African American 12 (12%); Asian, 27 (27%); and Others, 3 (3%)	0.05
Systolic BP (mm Hg)	127.8 ± 14.1	120.2 ± 17.5	0.003
Diastolic BP (mm Hg)	78.8 ± 10.0	78.7 ± 14.2	0.99
Duration of T2DM (years)	10.9 ± 8.2	–	
HbA1c	7.1±1.3 % (54.1±14.2 mmol/mol)	–	
HDL cholesterol levels (mg/dL)	51.8±18.2 (n=47)		
BAI	5.0 ± 4.3	2.7 ± 4.3	<0.001
BDI-II	6.6 ± 5.1	3.4 ± 5.1	<0.001
Total MoCA scores	25.9 ± 2.4	27.1 ± 2.4	0.002
MoCA: visuospatial	4.2 ± 0.8	4.5 ± 0.8	0.01
MoCA: naming	2.9 ± 0.2	3.0 ± 0.2	0.37
MoCA: attention	5.1 ± 0.9	5.5 ± 0.9	0.005
MoCA: language	2.2 ± 0.8	2.6 ± 0.8	0.002
MoCA: abstraction	1.9 ± 0.3	2.0 ± 0.3	0.05
MoCA: delayed Recall	3.4 ± 1.5	3.5 ± 1.5	0.80
MoCA: orientation	6.0 ± 0.2	6.0 ± 0.2	0.19

*T2DM* Type 2 diabetes mellitus, *SD* Standard deviation, *BMI* Body mass index, *BP* Blood pressure, *BDI-II* Beck depression inventory II, *BAI* Beck anxiety inventory, *MoCA* Montreal cognitive assessment.

### Regional MD changes in type 2 diabetes patients

**Figure 1:**
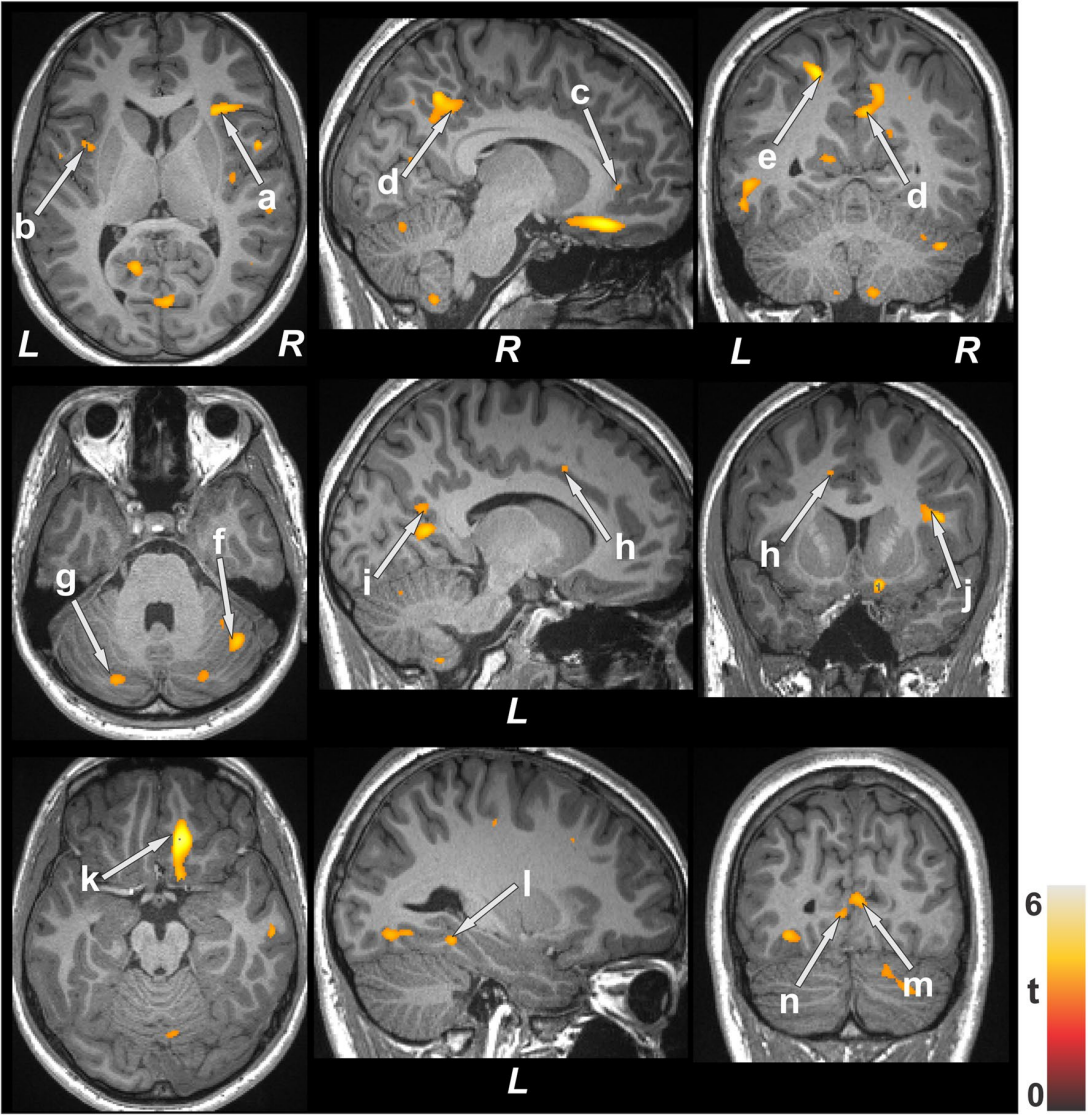
Brain sites with increased regional mean diffusivity (MD) values in patients with type 2 diabetes compared to control subjects. Brain regions showed increased MD values in the bilateral insula (**a**, **b**), bilateral anterior (**c**, **h**), and posterior (**d**, **i**) cingulate, left superior parietal cortices (**e**), bilateral cerebellum (**f**, **g**), right inferior frontal cortices (**j**), right prefrontal cortices (**k**), left para-hippocampal gyrus (**l**), and bilateral lingual gyrus (**m**, **n**).

**Figure 2:**
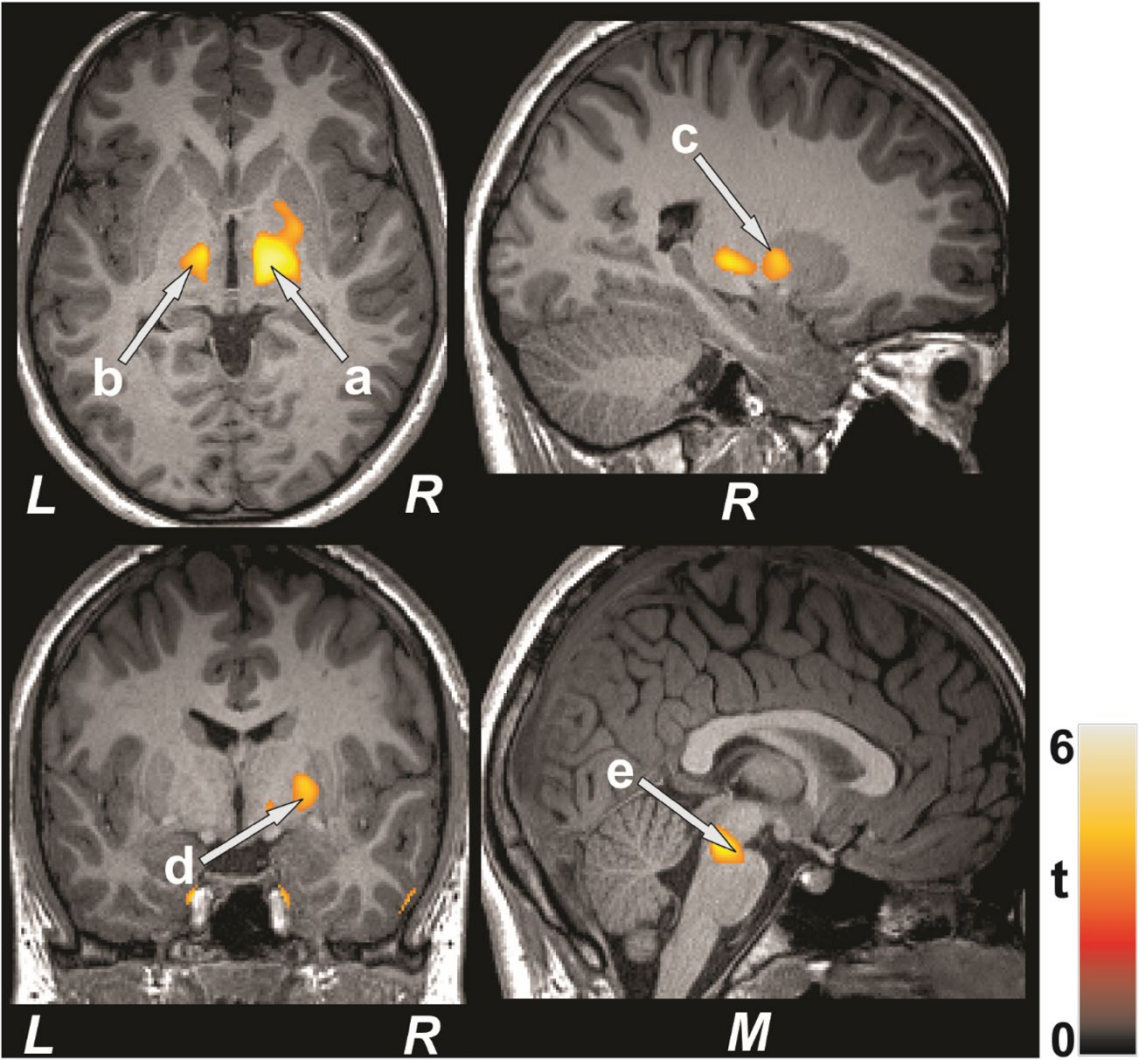
Brain regions with reduced regional MD values in type 2 diabetes compared to control subjects. These sites with reduced MD values included the bilateral thalamus (**a**, **b**), right putamen (**c**), right pallidum (**d**), and pons (**e**).

Multiple brain areas showed increased regional MD values, indicating chronic tissue changes, in type 2 diabetes patients compared to controls (Fig. 1), including the bilateral cerebellum, cerebellar vermis, left para-hippocampal gyrus, bilateral anterior and right posterior insula, and bilateral mid and right inferior frontal, and right prefrontal cortices. In addition, MD values were remarkably increased in the bilateral anterior, and posterior cingulate, left inferior, right superior, and bilateral mid temporal gyrus, left superior parietal cortices, bilateral lingual gyrus, and bilateral pre- and post-central gyri. Few brain regions showed decreased MD values, suggesting acute tissue changes, and included the bilateral thalamus, right putamen, pons, and right pallidum (Fig. 2). Regional brain MD values from sites showing significant differences between groups are summarized in Table 2.

**Table 2:** Regional brain mean diffusivity values (mean ± SD; Unit, ×10^−3^ mm^2^/s) of T2DM patients and control subjects.

Brain regions	Sites with increased MD values in T2DM over controls		
	T2DM (n = 68)	Control (n = 101)	*P* values
Left cerebellar cortex	0.94±0.08	0.88±0.08	<0.001
Right cerebellar cortex	0.90±0.06	0.84±0.06	<0.001
Cerebellar vermis	1.10±0.10	1.00±0.10	<0.001
Left anterior insula	0.98±0.07	0.93±0.07	<0.001
Right anterior insula	0.97±0.08	0.92±0.08	<0.001
Right posterior insula	1.32±0.14	1.24±0.14	<0.001
Right prefrontal cortex	0.86±0.05	0.82±0.05	<0.001
Left anterior cingulate	0.82±0.05	0.79±0.05	<0.001
Right anterior cingulate	0.88±0.05	0.85±0.05	<0.001
Left posterior cingulate	1.07±0.10	1.01±0.10	<0.001
Right posterior cingulate	1.00±0.09	0.95±0.09	<0.001
Left Parahipp Gyrus	0.89±0.07	0.86±0.07	<0.001
Left lingual gyrus	1.12±0.08	1.07±0.08	<0.001
Right lingual gyrus	1.17±0.09	1.11±0.09	<0.001
Left precentral gyrus	0.96±0.07	0.91±0.07	<0.001
Right precentral gyrus	0.99±0.09	0.94±0.09	<0.001
Left postcentral gyrus	0.95±0.07	0.91±0.07	<0.001
Right postcentral gyrus	0.94±0.07	0.90±0.07	<0.001
Right inf frontal gyrus	0.96±0.10	0.90±0.10	<0.001
Left inf temp gyrus	0.91±0.06	0.87±0.06	<0.001
Left mid frontal cortex	1.14±0.15	1.04±0.15	<0.001
Right mid frontal cortex	0.93±0.10	0.87±0.10	<0.001
Left mid temp gyrus	0.86±0.04	0.83±0.04	<0.001
Right mid temp gyrus	0.95±0.06	0.91±0.06	<0.001
Left sup parietal cortex	1.11±0.15	1.01±0.15	<0.001
Right sup temp cortex	0.96±0.07	0.92±0.07	<0.001
	Areas with reduced MD values in T2DM over controls		
Left thalamus	0.63±0.04	0.66±0.04	<0.001
Right thalamus	0.66±0.04	0.69±0.04	<0.001
Right putamen	0.64±0.05	0.67±0.05	<0.001
Right pallidum	0.60±0.07	0.65±0.07	<0.001
Pons	0.73±0.08	0.79±0.08	<0.001

*SD* Standard deviation, *T2DM* Type 2 diabetes mellitus, *Parahipp* Para-hippocampal, *Inf* Inferior, *Mid* Middle, *Temp* Temporal, *Sup* Superior.

### Correlations between MD and BDI-II, BAI, MoCA scores in type 2 diabetes patients

Depression (BDI-II) scores showed positive associations with MD values at the right prefrontal cortices, left inferior, bilateral mid, and right superior temporal gyrus, left mid and right inferior frontal cortices, left superior parietal cortices, right lingual gyrus, suggesting that the regions that showed chronic tissue changes are associated with higher depressive symptoms. Brain regions that showed decreased MD values in type 2 diabetes patients included the thalamus, putamen, and pons, and correlated with negative association with BDI-II scores, suggesting that the regions with acute tissue changes are related to depressive symptoms. Positive relationships were observed in the right mid and inferior frontal cortices, right anterior cingulate, bilateral mid temporal gyrus, and bilateral lingual gyrus; negative associations in pons between anxiety scores and MD values among the regions that showed altered MD values. MoCA scores were negatively correlated with MD values at the bilateral mid frontal, left superior parietal cortices, bilateral pre- and left post-central gyrus, and positively correlated with the thalamus. MoCA sub-scores, including the visuospatial function, language, attention, and abstraction were negatively associated with MD values from the cerebellar cortices, prefrontal cortices, parahippocampal gyrus, lingual gyrus, frontal, parietal, and temporal cortices, pre- and post-central gyrus, and positively correlated with the thalamus and putamen. The correlation coefficients for sites showing significant associations are summarized in Tables 3 and 4, which are significant based on the Benjamini-Hochberg procedures with false discovery rate of 0.05.

**Table 3:** Correlation between regional mean diffusivity values and mood and cognition in type 2 diabetes subjects.

Variables	Associations	Brain regions	Correlation coefficients (r)	*P* values
BDI-II vs MD values	Positive	Right prefrontal cortices	0.36	0.003
		Right lingual gyrus	0.32	0.009
		Left mid frontal cortices	0.32	0.008
		Right inferior frontal cortices	0.34	0.005
		Left inferior temporal gyrus	0.36	0.003
		Left mid temporal gyrus	0.36	0.003
		Right mid temporal gyrus	0.36	0.003
		Right superior temporal gyrus	0.36	0.003
		Left superior parietal cortices	0.37	0.003
	Negative	Left thalamus	− 0.33	0.008
		Right putamen	− 0.37	0.003
		Pons	− 0.39	0.001
BAI vs MD values	Positive	Left anterior cingulate	0.36	0.003
		Right anterior cingulate	0.35	0.004
		Left lingual gyrus	0.32	0.008
		Right lingual gyrus	0.34	0.005
		Right mid frontal cortices	0.35	0.004
		Right inferior frontal cortices	0.37	0.003
		Left mid temporal gyrus	0.40	<0.001
		Right mid temporal gyrus	0.35	0.004
	Negative	Pons	− 0.40	<0.001
MoCA vs MD values	Negative	Left mid frontal cortices	− 0.37	0.002
		Right mid frontal cortices	− 0.33	0.006
		Left superior parietal cortices	− 0.36	0.003
		Left precentral gyrus	− 0.36	0.003
		Right precentral gyrus	− 0.38	0.002
		Left postcentral gyrus	− 0.36	0.003
	Positive	Left thalamus	0.35	0.004
		Right thalamus	0.33	0.008

*BDI-II* Beck depression inventory II, *MD* Mean diffusivity, *BAI* Beck anxiety inventory, *MoCA* Montreal cognitive assessment.

**Table 4:** Correlations between regional brain mean diffusivity values and MoCA sub-scores in T2DM adults.

Variables	Associations	Brain regions	Correlation coefficients (r)	*P* values
Visuosp vs MD values	Negative	Left cerebellar cortex	− 0.35	0.004
		Right cerebellar cortex	− 0.34	0.005
		Left inf temp gyrus	− 0.36	0.003
		Left lingual gyrus	− 0.33	0.007
		Left mid frontal cortices	− 0.39	0.001
		Left mid temp gyrus	− 0.32	0.008
		Right mid temp gyrus	− 0.43	<0.001
		Right precentral gyrus	− 0.37	0.002
		Left postcentral gyrus	− 0.33	0.006
		Left sup parietal cortices	− 0.35	0.005
Lang vs MD values	Negative	Left cerebellar cortex	− 0.39	0.001
		Right cerebellar cortex	− 0.43	<0.001
		Left inf temp gyrus	− 0.33	0.008
		Left mid frontal cortices	− 0.35	0.004
		Right mid frontal cortices	− 0.33	0.006
		Left mid temp gyrus	− 0.39	0.001
		Right mid temp gyrus	− 0.33	0.007
		Right precentral gyrus	− 0.32	0.009
		Left postcentral gyrus	− 0.39	0.001
		Left sup parietal cortices	− 0.33	0.007
		Right sup temp gyrus	− 0.34	0.006
	Positive	Left thalamus	0.35	0.004
		Right thalamus	0.38	0.002
		Right putamen	0.36	0.003
Attn vs MD values	Negative	Right cerebellar cortex	− 0.37	0.002
		Right prefrontal cortex	− 0.37	0.002
		Left mid frontal cortices	− 0.41	<0.001
		Right mid frontal cortices	− 0.33	0.007
		Left mid temp gyrus	− 0.34	0.006
		Right inf frontal gyrus	− 0.38	0.002
		Left parahipp gyrus	− 0.33	0.008
		Left precentral gyrus	− 0.42	<0.001
		Left postcentral gyrus	− 0.36	0.003
		Right postcentral gyrus	− 0.33	0.007
		Right sup temp gyrus	− 0.36	0.003
	Positive	Left thalamus	0.35	0.005
Abstr vs MD values	Neg	Right cerebellar cortex	− 0.37	0.002
		Left anterior cingulate	− 0.34	0.005
		Right anterior cingulate	− 0.37	0.003
		Right prefrontal cortex	− 0.40	<0.001
		Right lingual gyrus	− 0.35	0.004
		Left mid frontal cortices	− 0.44	<0.001
		Right mid frontal cortices	− 0.45	<0.001
		Left mid temp gyrus	− 0.32	0.009
		Right mid temp gyrus	− 0.35	0.004
		Right inf frontal gyrus	− 0.37	0.002
		Left precentral gyrus	− 0.34	0.006
		Left postcentral gyrus	− 0.34	0.006

Visuosp = *MoCA* Visuospatial, *Neg* Negative, *Inf* Inferior, *Sup* Superior; Lang = *MoCA* Language, *Pos* Positive, Attn = *MoCA* Attention, *Parahipp* Parahippocampal, Abstr = *MoCA* Abstraction.

## Discussion

In the current study, we found that people living with type 2 diabetes mellitus showed widespread microstructural disruptions in various brain regions regulating cognition and mood functions compared to control subjects; these tissue changes were predominantly in chronic stages, with few sites in the acute stage. Congruent with study findings, overall cognitive deficits were observed in patients with type 2 diabetes, with impairment in visuospatial, attention, and language cognitive sub-domains. Depression and anxiety scores were significantly increased in type 2 diabetes patients over healthy controls, and significant associations were observed between microstructural tissue integrity of mood and cognitive regulatory sites and those symptoms, which is a first report in type 2 diabetes mellitus, based on our best knowledge. Considering that microstructural tissue impairment underlies common mechanisms of cognitive and mood dysfunction and disrupts the large-scale distributed brain cognitive and mood regulatory networks, our findings imply that extensive microstructural acute and chronic tissue changes in those areas play a distinct role in cognition and mood deficits in type 2 diabetes condition. Our investigation provides insight into the nature and extent of microstructural neuropathological changes on mood and cognition functions in type 2 diabetes mellitus.

Patients with type 2 diabetes showed extensive disruptions in the microstructural tissue integrity in areas predominantly located in the frontal, temporal, and parietal cortices, and subcortical regions reinforced by decreased nodal efficiency of these sites based on topological network analysis^17^ Several studies have shown microstructural tissue changes in the para-hippocampal gyrus, insular, prefrontal, and parietal cortices^18^ as was also demonstrated in our study with widespread changes in those areas with major sites showing chronic changes and a few regions with acute tissue injury. Brain fiber connectivity analyses from previous studies showed reduced connectivity in both cerebellar and cerebro-cerebellar circuits in patients with type 2 diabetes relative to control subjects^15^ These encompassed fibers connecting the cerebellum to vermis, and anterior crus to cortical areas, including the precentral, frontal, and superior parietal gyri, the regions that showed chronic tissue changes with increased MD values in type 2 diabetes patients in our study.

Previous studies have demonstrated that adults with type 2 diabetes have an increase in cognitive deficits compared to healthy controls in multiple domains, including attention, psychomotor efficiency, executive function, verbal/emotional/working memory, and information processing speed^17,18^ Our study showed similar findings with deficits in visuospatial processing, attention, and language domains. Prominent cognition and mild cognitive impairments are more likely to develop in individuals with type 2 diabetes, and the risks for Alzheimer's disease and vascular dementia are increased^19,20^ Reduced functional connectivity in patients with type 2 diabetes has been reported between regions of the default mode network, highly connected regions in the brain, including the frontal gyrus and medial temporal gyrus^21^ implicating an important role in global cognitive processing, memory, executive functioning, and processing speed. Such regions showed altered MD values and significant correlations with cognitive scores in type 2 diabetes patients in this study. Earlier studies have demonstrated that cognitive impairment in patients with type 2 diabetes is associated with more disruption in neural structures, including the fronto-temporal regions and thalamic radiations^22^ fewer white matter connections, and altered nodal network efficiency in temporal lobe^13,23^ these regions appeared to show abnormal MD values, which were significantly correlated with cognition scores in our study.

Depression and anxiety symptoms may result from difficulties in coping with chronic disease. However, the chronic metabolic consequences of type 2 diabetes may disturb cerebral neurotransmitters and impair brain tissue, thus predisposing people to depressive and anxiety symptoms^24^ In addition, mood changes in patients with type 2 diabetes might result from brain vascular damage^25,26^ On the other hand, the relationships between type 2 diabetes and mood deficits may be bidirectional, as these mood changes may even further contribute to enhanced brain tissue changes in patients with type 2 diabetes^27^ Neuroimaging studies have identified a consistently altered network of brain regions in anxious and depressed patients^28,29^ including the basal ganglia, prefrontal, and frontal cortices, anterior cingulate cortex, and thalamus, and these areas showed altered regional MD values and associations with BDI and BAI scores here. The cortico-striatal-pallidal-thalamic circuit, which comprises of neuroanatomical loops that connect various brain sites including anterior cingulate cortex, prefrontal cortex, basal ganglia and thalamus in a highly organized and integrated manner, supports diverse cognitive and emotional processes that are shown dysfunctional in depressed patients. In this study, we observed aberrant MD changes and correlations with BDI-II and BAI scores in these regions in patients with type 2 diabetes, which might be suggestive of bidirectional changes for patients with higher mood deficits.

Although the underlying mechanisms responsible for tissue changes in cognitive and mood regulatory areas contributing to functional deficits in type 2 diabetes mellitus are not fully elucidated, they may include hyperglycemia, insulin resistance, oxidative stress, and neuroinflammation-induced processes^30,31^ In addition, other common causes for depressive symptoms in type 2 diabetes may include lack of physical exercise, poor sleep, and diet changes, a common pathway that could activate and disturb the stress system, contributing to further brain tissue changes in cognition and mood regulatory areas. Chronic depressive and anxiety symptoms, components of stress leads to increased production of cortisol levels and inflammatory cytokines due to activated sympathetic nervous system (SNS), which is an integrative system that reacts to stress and a component of the autonomic nervous system and activated hypothalamus-pituitary-adrenal axis^32^ Glucocorticoid receptors are distributed in the hippocampus, hypothalamus, and other regions^33^ and increased cortisol levels can further contribute to tissue changes in those areas^34,35^ In addition, prolonged SNS activation and chronic hypercortisolemia elevate insulin resistance, visceral obesity, and lead to the metabolic syndrome; all factors leading to oxidative stress and inflammatory response contributing to type 2 diabetes mellitus. Also, chronic stress induced inflammatory responses interact with neurotransmitter metabolism, neuroendocrine function, synaptic plasticity, and behavior, and activate the fear system emerging anxiety and depression symptoms.

One of the limitations of our study is that some control subjects (50%) self-confirmed that they did not have type 2 diabetes, which may have contaminated data diluting findings. However, although their HbA1c levels were not available, those control subjects had normal cognitive and mood functions indicating very less influence, if any, in this study. Also, some of our T2DM adults had retinopathy, neuropathy, and hypertension, and the brain tissue changes observed in our study might be impacted with these comorbidities, and need to be explored in future with larger sample size.

## Conclusions

In conclusion, people living with type 2 diabetes condition show widespread microstructural chronic brain tissue changes primarily in sites that mediate mood and cognition functions, and tissue integrity in those areas correlate with mood and cognitive symptoms. The data suggest that type 2 diabetes mellitus leads to an alteration in the microstructural chronic and acute cerebral tissue changes that may account for the observed impaired mood and cognition. The findings indicate that MRI-based biomarkers, such as MD, can be used for early detection of brain tissue changes in cognitive and mood regulatory regions in patients with type 2 diabetes.

## Materials and methods

We recruited 68 people with type 2 diabetes mellitus from the University of California Los Angeles (UCLA) Gonda Diabetes Center, as well as from the surrounding community and all type 2 diabetes patients were on medications. 101 healthy, non-diabetic controls were recruited through flyer advertisement at the UCLA campus and the West Los Angeles area. Demographic, physiologic, mood, and cognitive data are summarized in Table 1. Inclusion criteria for type 2 diabetes patients included ages 40-65 years, on stable medications, and able to lay supine for MRI. Most of the control subjects were assessed for HbA_1_c levels using the point-of-care tests utilizing fingerstick blood, and some controls self-confirmed normoglycemia. Control subjects were 40-65 years of age, without hypertension, and diabetes mellitus. The exclusion criteria for type 2 diabetes patients and control subjects included psychiatric disease (clinical depression, schizophrenia, manic-depressive), diagnosed brain condition (seizure disorder, head trauma), history of stroke, heart failure, airway or chest deformities that would interfere with breathing, mechanical ventilator support, and renal failure (requiring dialysis). Subjects were also excluded if there was presence of sleep disordered breathing, dementia, cystic fibrosis, chronic obstructive pulmonary disease, brain mass lesions, or dependency on drugs (e.g., tobacco or cocaine use) that would introduce brain tissue changes, or the presence of claustrophobia, body weight more than 160 kg (limitations of scanner table), or metallic implants. The Diabetes Complications Severity Index (DCSI) was assessed from each patient’s clinical record to document complications including retinopathy, nephropathy, neuropathy, cerebrovascular, cardiovascular, and peripheral vascular disease. Written informed consent was obtained from all subjects prior to the study; the UCLA Institutional Review Board approved the entire protocol. All methods were performed in accordance with the relevant guidelines and regulations.

### Depression and anxiety assessment

The Beck Anxiety Inventory (BAI)^36^ was administered in type 2 diabetes patients and control subjects to evaluate anxiety symptoms. Depression was measured by self-reported questionnaire using the Beck Depression Inventory (BDI-II)^37^ These self-administered questionnaires consisted of 21 questions per inventory, with scores ranging from 0-3 for each question and a total score for each inventory ranging from 0-63, depending on the severity of symptoms. Type 2 diabetic patients or healthy control subjects with BDI-II or BAI values > 9 were considered to have symptoms of depression or anxiety, respectively.

### Cognition assessment

We administered Montreal Cognitive Assessment (MoCA)^38^ on patients with type 2 diabetes and control subjects. Multiple aspects of cognition, including visuospatial skills, attention and focus, executive function, concentration, delayed memory recall, naming, and language was assessed using the MoCA. The MoCA is widely used as a screening test for cognitive impairment, with scores ranging from 0 to 30 (≥ 26 normal).

### Magnetic resonance imaging

Brain imaging studies were performed using a 3.0-Tesla MRI scanner (Siemens, Magnetom Prisma, Erlangen, Germany). High-resolution T1-weighted images were collected using the magnetization-prepared rapid acquisition gradient-echo pulse sequence [repetition-time (TR) = 2200 ms; echo-time (TE) = 2.4 ms; inversion time = 900 ms; flip angle (FA) = 9°; matrix size = 320 × 320; field-of-view (FOV) = 230 × 230 mm^2^; slice thickness = 0.9 mm)]. Proton-density (PD) and T2-weighted images (TR = 10,000 ms; TE1, 2 = 17, 134 ms; FA = 130°; matrix size = 256 × 256; FOV = 230 × 230 mm^2^; slice thickness = 3.5 mm) were collected using a dual-echo turbo spin-echo sequence in the axial plane. DTI data were collected using a single-shot echo planar imaging with twice-refocused spin-echo pulse sequence (TR = 12,200 ms; TE = 87 ms; flip angle = 90°; bandwidth = 1,345 Hz/pixel; matrix size = 128×128; FOV = 230×230 mm; slice thickness = 1.7 mm, diffusion values = 0 and 800 s/mm^2^, diffusion directions = 30, separate series = 2). The parallel imaging technique, generalized auto-calibrating partially parallel acquisition (GRAPPA), with an acceleration factor of two, was used for DTI data collection.

### Data processing

This study used the SPM12 software package (Department of Cognitive Neurology, Wellcome, UK), Diffusion Toolkit (TrackVis.org, Massachusetts General Hospital, USA), MRIcroN, and MATLAB-based software for data processing, analysis, and visualization. We performed visual assessment of T1-, T2-, and PD-weighted images of all subjects for any major pathology (e.g., cystic lesion, infarct, or tumor) and subsequently excluded subjects if any abnormalities were found. Diffusion and non-diffusion weighted images of all type 2 diabetes patients and healthy control subjects were also assessed for any head-motion related or other imaging artifacts before quantifying MD maps.

### MD calculation & processing

Diffusion tensor matrices were quantified with the Diffusion Toolkit software using diffusion-weighted (b=800 s/mm^2^) and non-diffusion weighted images (b=0 s/mm^2^)^39,40^ The diffusion tensor matrices were diagonalized, and principal eigenvalues (λ_1_, λ_2_, and λ_3_) were calculated. The principal eigenvalues were then used to calculate MD [MD = (λ_1_+λ_2_+λ_3_)/3] values at each voxel, with voxel intensities on the MD maps showing the corresponding diffusion values. A fixed threshold value was used to mask-out background noise and non-brain regions on MD maps in Diffusion Toolkit.

We used the MATLAB-based SPM12 software for pre-processing of the MD maps. The MD maps, derived from each DTI series, were realigned to remove any potential variation from head motion and averaged. Similarly, non-diffusion weighted images were also realigned and averaged. The averaged MD maps were normalized to Montreal Neurological Institute (MNI) space. Non-diffusion weighted (b0) images were normalized to MNI space using a unified segmentation approach^41^ and the resulting normalization parameters were applied to corresponding MD maps. The normalized MD maps were smoothed with a Gaussian filter (8 mm).

### Statistical analyses

We used the statistical package for social sciences (SPSS V28) for assessment of demographic, physiological, mood, and cognitive data. The numerical demographic and clinical variables were compared between groups with independent samples t-tests, and categorical characteristics were compared using the Chi-square test. Mood and cognition data were compared between type 2 diabetes patients and control subjects using ANCOVA (covariates, age and sex). Statistical threshold values of *p*<0.05 were considered as significant differences.

For regional brain MD differences between type 2 diabetes patients and control subjects, the smoothed MD maps were compared voxel-by-voxel using ANCOVA (SPM12; covariates, age and sex; false discovery rate, *p*<0.05). The statistical parametric maps showing brain sites with significant MD differences between groups were superimposed onto a mean anatomical image for structural identification using MRIcroN software. Regions of interest (ROI) values were obtained from brain regions with significant differences between T2DM and controls (ANCOVA, SPSS, covariates, age and sex, Bonferroni corrected).

Whole-brain MD maps were correlated voxel-by-voxel with mood and cognition scores including BAI, BDI-II, MoCA, and MoCA sub-scores in type 2 diabetes patients using partial correlations (SPM12; covariates, age and sex; *p*<0.005). The ROI values were obtained from brain sites showing significant associations with mood and cognition values. The Benjamini-Hochberg method was used to determine if the associations survived for multiple corrections, controlling for the false discovery rate with *p*<0.05.

## Data Availability

The data that support the findings of this study are available on request from the corresponding author. The data are not publicly available due to privacy or ethical restrictions.

## Abbreviation

BAI: Beck anxiety inventory
BDI-II: Beck depression inventory
DTI: Diffusion tensor imaging
FA: Flip angle
FOV: Field-of-view
MD: Mean diffusivity
MNI: Montreal neurological Institute
MoCA: Montreal cognitive assessment
PD: Proton-density
ROI: Region of interest
SNS: Sympathetic nervous system
SPM12: Statistical parametric mapping package
SPSS V28: Statistical package for social sciences
TR: Repetition-time
TE: Echo-time

## References

[1] Cukierman T, Gerstein HC, Williamson JD (2005). Cognitive decline and dementia in diabetes–systematic overview of prospective observational studies. Diabetologia.

[2] Anderson RJ, Freedland KE, Clouse RE, Lustman PJ (2001). The prevalence of comorbid depression in adults with diabetes: A meta-analysis. Diabetes care.

[3] Nichols GA, Brown JB (2003). Unadjusted and adjusted prevalence of diagnosed depression in type 2 diabetes. Diabetes Care.

[4] Cheng G, Huang C, Deng H, Wang H (2012). Diabetes as a risk factor for dementia and mild cognitive impairment: A meta-analysis of longitudinal studies. Int. Med. J..

[5] Exalto LG, Whitmer RA, Kappele LJ, Biessels GJ (2012). An update on type 2 diabetes, vascular dementia and Alzheimer's disease. Exp. Gerontol..

[6] Peila R, Rodriguez BL, Launer LJ, Honolulu-Asia Aging S (2002). Type 2 diabetes, APOE gene, and the risk for dementia and related pathologies: The honolulu-asia aging study. Diabetes.

[7] Crane PK (2013). Glucose levels and risk of dementia. N. Engl. J. Med..

[8] Egede LE, Zheng D, Simpson K (2002). Comorbid depression is associated with increased health care use and expenditures in individuals with diabetes. Diabetes Care.

[9] Holt RI, Katon WJ (2012). Dialogue on diabetes and depression: dealing with the double burden of co-morbidity. J. Affect. Disord..

[10] Egede LE, Walker RJ, Bishu K, Dismuke CE (2016). Trends in costs of depression in adults with diabetes in the United States: Medical expenditure panel survey, 2004–2011. J. Gen. Intern. Med..

[11] Grigsby AB, Anderson RJ, Freedland KE, Clouse RE, Lustman PJ (2002). Prevalence of anxiety in adults with diabetes: A systematic review. J. Psychosom. Res..

[12] Tovilla-Zarate C (2012). Prevalence of anxiety and depression among outpatients with type 2 diabetes in the Mexican population. PLoS One.

[13] van Bussel FC (2016). Altered hippocampal white matter connectivity in type 2 diabetes mellitus and memory decrements. J. Neuroendocrinol..

[14] Zhang A (2013). White matter tract integrity of anterior limb of internal capsule in major depression and type 2 diabetes. Neuropsychopharmacology.

[15] Fang P (2017). Changes in the cerebellar and cerebro-cerebellar circuit in type 2 diabetes. Brain Res. Bull..

[16] Nouwen A (2017). Microstructural abnormalities in white and gray matter in obese adolescents with and without type 2 diabetes. Neuroimage. Clin..

[17] Zhang J (2016). Disrupted white matter network and cognitive decline in type 2 diabetes patients. J. Alzheimers Dis..

[18] Yau PL, Kluger A, Borod JC, Convit A (2014). Neural substrates of verbal memory impairments in adults with type 2 diabetes mellitus. J. Clin. Exp. Neuropsychol..

[19] Luchsinger JA (2007). Relation of diabetes to mild cognitive impairment. Arch. Neurol..

[20] Yaffe K (2004). Diabetes, impaired fasting glucose, and development of cognitive impairment in older women. Neurology.

[21] Musen G (2012). Resting-state brain functional connectivity is altered in type 2 diabetes. Diabetes.

[22] Xiong Y (2016). A diffusion tensor imaging study on white matter abnormalities in patients with type 2 diabetes using tract-based spatial statistics. AJNR Am. J. Neuroradiol..

[23] Zhang Y (2019). Altered brain structural topological properties in type 2 diabetes mellitus patients without complications. J. Diabetes.

[24] Krabbe KS (2007). Brain-derived neurotrophic factor (BDNF) and type 2 diabetes. Diabetologia.

[25] Baldwin RC, O'Brien J (2002). Vascular basis of late-onset depressive disorder. Br. J. Psychiatry: J. Mental Sci..

[26] Bruce DG (2006). Vascular depression in older people with diabetes. Diabetologia.

[27] Knol MJ (2006). Depression as a risk factor for the onset of type 2 diabetes mellitus A meta-analysis. Diabetologia.

[28] Holzschneider K, Mulert C (2011). Neuroimaging in anxiety disorders. Dialogues Clin. Neurosci..

[29] Kanner AM (2004). Structural MRI changes of the brain in depression. Clin. EEG Neurosci..

[30] Feinkohl I, Price JF, Strachan MW, Frier BM (2015). The impact of diabetes on cognitive decline: Potential vascular, metabolic, and psychosocial risk factors. Alzheimers Res. Ther..

[31] Geijselaers SLC, Sep SJS, Stehouwer CDA, Biessels GJ (2015). Glucose regulation, cognition, and brain MRI in type 2 diabetes: A systematic review. Lancet. Diabetes Endocrinol..

[32] Kyrou I, Tsigos C (2009). Stress hormones: Physiological stress and regulation of metabolism. Curr. Opin. Pharmacol..

[33] Alexis MN, Stylianopoulou F, Kitraki E, Sekeris CE (1983). The distribution and properties of the glucocorticoid receptor from rat brain and pituitary. J. Biol. Chem..

[34] Sapolsky RM, Uno H, Rebert CS, Finch CE (1990). Hippocampal damage associated with prolonged glucocorticoid exposure in primates. J. Neurosci: Off. J. Soc. Neurosci..

[35] Sheline YI (2003). Neuroimaging studies of mood disorder effects on the brain. Biol.Psychiatry.

[36] Beck AT, Epstein N, Brown G, Steer RA (1988). An inventory for measuring clinical anxiety: Psychometric properties. J. Consult. Clin. Psychology.

[37] Beck AT, Steer RA, Ball R, Ranieri W (1996). Comparison of Beck Depression Inventories -IA and -II in psychiatric outpatients. J. Pers. Asses..

[38] Nasreddine ZS (2005). The montreal cognitive assessment, MoCA: A brief screening tool for mild cognitive impairment. J. Am. Geriatr. Soc..

[39] Basser PJ, Pierpaoli C (1998). A simplified method to measure the diffusion tensor from seven MR images. Magn. Reson. Med..

[40] Pierpaoli C, Jezzard P, Basser PJ, Barnett A, Di Chiro G (1996). Diffusion tensor MR imaging of the human brain. Radiology.

[41] Ashburner J, Friston KJ (2005). Unified segmentation. Neuroimage.

